# Postoperative Delirium After Cardiac Surgery: Psychiatric Vulnerability, Biological Mechanisms, and Prevention Strategies

**DOI:** 10.3390/medsci14020176

**Published:** 2026-03-31

**Authors:** Vasileios Leivaditis, Anastasios Sepetis, Francesk Mulita, Athanasios Papatriantafyllou, Sofoklis Mitsos, Periklis Tomos, Konstantinos Grapatsas, Ejona Shaska, Elias Liolis, Efstratios Koletsis, Nikolaos Baikoussis

**Affiliations:** 1 Department of Cardiothoracic and Vascular Surgery, Westpfalz Klinikum, 67655 Kaiserslautern, Germany; thanospap9@yahoo.gr; 2 Postgraduate Health and Social Care Management Program, Department of Business Administration, University of West Attica, 12244 Athens, Greece; tsepet@uniwa.gr; 3 Department of General Surgery, General Hospital of Eastern Achaia—Unit of Aigio, 25100 Aigio, Greece; 4 Department of Thoracic Surgery, Attikon General Hospital, National and Kapodistrian University of Athens, 12462 Athens, Greece; sophocmit@yahoo.gr (S.M.); periklistomos@hotmail.com (P.T.); 5 Department of Thoracic Surgery and Thoracic Endoscopy, Ruhrlandklinik, West German Lung Center, University Hospital Essen, University Duisburg-Essen, 45141 Essen, Germany; grapatsaskostas@gmail.com; 6 Department of Psychiatry, “Ali Mihali” Psychiatric Hospital, 9401 Vlora, Albania; zilja.jona@yahoo.it; 7 Department of Oncology, General University Hospital of Patras, 26504 Patras, Greece; lioliselias@yahoo.gr; 8 Department of Cardiothoracic Surgery, General University Hospital of Patras, 26504 Patras, Greece; ekoletsis@hotmail.com; 9 Department of Cardiac Surgery, Ippokrateio General Hospital of Athens, 11527 Athens, Greece; nikolaos.baikoussis@gmail.com

**Keywords:** delirium, cardiac surgery, depression, cognitive impairment, neuroinflammation, dexmedetomidine, ICU, postoperative cognitive dysfunction

## Abstract

Introduction: Delirium is one of the most common and serious neuropsychiatric complications following cardiac surgery. It is associated with increased mortality, prolonged intensive care unit (ICU) and hospital stay, long-term cognitive decline, and reduced quality of life. Aims and Objectives: The aim of this study is to synthesize current evidence on the epidemiology, psychiatric and psychosocial risk factors, biological mechanisms, perioperative modifiers, prevention strategies, and long-term outcomes of delirium after cardiac surgery, with particular emphasis on its role as a marker of brain vulnerability. Materials and Methods: A narrative literature review was conducted using articles published between 1990 and 2025, identified through the PubMed and ScienceDirect databases. The search strategy included the terms “delirium,” “cardiac surgery,” “psychiatric disorders,” and “cognitive impairment.” Results: Recent evidence suggests that approximately one quarter of patients undergoing cardiac surgery develop delirium, with hypoactive forms frequently remaining underrecognized in clinical practice. Pre-existing depression, cognitive impairment, substance use disorders, low educational level, frailty, and social isolation significantly increase the risk of postoperative delirium. Within a stress–diathesis framework, peripheral physiological insults may be reflected centrally as acute brain dysfunction in vulnerable individuals. Modifiable perioperative factors include sedative choice and depth, as well as sleep and circadian disruption. Multicomponent non-pharmacological interventions, early mobilization, structured psychiatric and cognitive screening, and dexmedetomidine-based sedation have demonstrated consistent efficacy in reducing the incidence and/or duration of delirium. Furthermore, delirium has been associated with persistent cognitive and psychiatric morbidity, functional decline, and increased long-term mortality. Conclusions: Delirium following cardiac surgery is a multifactorial syndrome with significant short- and long-term consequences. A comprehensive, multidisciplinary approach integrating biological, psychiatric, and perioperative perspectives is essential for effective risk stratification, prevention, and long-term follow-up.

## 1. Introduction

### 1.1. Clinical Significance of Postoperative Delirium in Cardiac Surgery

Postoperative delirium is an acute disturbance in attention and awareness that develops over a short period of time, represents a change from baseline, and tends to fluctuate in severity during the course of the day [1,2]. According to the Diagnostic and Statistical Manual of Mental Disorders, Fifth Edition (DSM-5), this syndrome also involves cognitive dysfunctions such as amnesia, disorientation, language impairment, or perceptual disturbances, and must not be attributable to a pre-existing neurocognitive disorder but rather to an underlying medical condition [1].

Delirium is one of the most frequent and serious complications of cardiac surgery. Studies employing validated screening instruments estimate its incidence to range from 26% to 52%, while a recent large-scale systematic review and meta-analysis of 106 studies reported a weighted pooled incidence of 23% [2,3]. Variability in reported incidence is largely attributed to differences in assessment methodology, timing, and screening tools, with less rigorous clinical detection methods likely underestimating the true burden [2].

Postoperative delirium following cardiac surgery has important clinical ramifications that extend well beyond the acute hospitalization phase. It has been independently associated with increased in-hospital and long-term mortality, as well as prolonged intensive care unit (ICU) and hospital stay, higher readmission rates, increased healthcare costs, reduced functional status, and persistent cognitive decline [2,4,5]. These adverse outcomes have been consistently demonstrated across multiple studies, with evidence indicating that affected patients may exhibit sustained cognitive impairment for months after surgery, and in many cases fail to return to their preoperative baseline [6].

Increasingly, delirium is conceptualized as a manifestation of “brain frailty,” reflecting reduced neural resilience to physiological stress. The substantial human and economic burden associated with postoperative delirium underlines the importance of a comprehensive understanding of its risk factors, underlying mechanisms, and prevention strategies.

### 1.2. Why Cardiac Surgery Is a High-Risk Setting

Cardiac procedures represent a particularly high-risk setting for the development of postoperative delirium. The pathogenesis of delirium in this context is multifactorial and includes several mechanisms that are unique to cardiac surgery. The use of cardiopulmonary bypass (CPB) induces a non-physiological state that triggers a pronounced systemic inflammatory response. Exposure of blood to the artificial surfaces of the CPB circuit activates the complement cascade, promotes cytokine release, and leads to leukocyte activation [7,8].

This inflammatory response may compromise the integrity of the blood–brain barrier (BBB), allowing circulating inflammatory mediators and neurotoxins to access the central nervous system [9]. In addition, the generation of gaseous and particulate microemboli during CPB can result in their embolization within the cerebral vasculature, leading to focal ischemia and neuronal injury [10,11].

Cerebral hypoperfusion during bypass represents another important mechanism of brain injury, particularly in patients with impaired cerebrovascular autoregulation [12]. Furthermore, patients undergoing cardiac surgery with CPB are typically older and have a higher burden of comorbidities compared to those not requiring bypass. Conditions such as hypertension, diabetes mellitus, cerebrovascular disease, and chronic kidney disease are well-established predisposing factors for delirium [13,14].

More complex procedures, such as combined valve and coronary surgery, are associated with prolonged operative and CPB times, thereby increasing both the duration of exposure to non-physiological conditions and the embolic load [15]. Collectively, these factors create a “perfect storm” of predisposing vulnerabilities and precipitating insults, placing the cardiac surgical population at a uniquely elevated risk for postoperative delirium.

### 1.3. Rationale and Scope of the Review

Although postoperative delirium after cardiac surgery is a well-recognized clinical entity, psychiatric and psychosocial risk factors remain underrecognized and are often inadequately addressed in perioperative management. Historically, risk stratification models in cardiac surgery have primarily focused on somatic and procedural variables, frequently overlooking the contribution of pre-existing psychiatric disorders [16].

This omission is clinically significant, as delirium is increasingly understood to arise from the interaction between baseline neuropsychiatric vulnerability and acute perioperative insults, as described by the stress–diathesis model [17,18]. Therefore, there is a need for integrated biological–psychiatric approaches that can improve risk stratification, guide targeted interventions, and ultimately reduce the incidence and impact of delirium in this high-risk population.

The present review aims to synthesize current evidence on the epidemiology, psychiatric and biological risk factors, pathophysiological mechanisms, and prevention strategies of delirium following cardiac surgery, with particular emphasis on underrecognized psychiatric and psychosocial contributors. In this review, the terms “brain vulnerability” and “brain frailty” are used to describe overlapping concepts referring to reduced neural reserve and resilience to physiological stress, as conceptualized within the stress–diathesis model.

## 2. Materials and Methods

### 2.1. Study Design

This article presents a narrative review synthesizing multidisciplinary evidence on postoperative delirium following cardiac surgery. A narrative approach was selected due to the heterogeneity of available studies in terms of definitions, patient populations, and delirium assessment methods, as well as the need to integrate psychiatric, neurological, anesthetic, and surgical perspectives. The review aimed to provide a comprehensive and integrative overview rather than an exhaustive systematic synthesis, with emphasis on clinically relevant and conceptually informative studies.

### 2.2. Data Sources

A comprehensive literature search was conducted using the PubMed/MEDLINE, Scopus, and Web of Science databases. The search included articles published between 1990 and December 2025, thereby covering the evolution of the field from early descriptive studies to more recent prospective cohort studies, randomized controlled trials, and meta-analyses.

### 2.3. Search Strategy

The search strategy employed a combination of Medical Subject Headings (MeSH) terms and free-text keywords, including “delirium,” “postoperative delirium,” “cardiac surgery,” “cardiopulmonary bypass,” “psychiatric disorders,” “depression,” “anxiety,” “cognitive impairment,” “neuroinflammation,” “prevention,” and “dexmedetomidine.” Boolean operators (AND, OR) were used to combine search terms and generate targeted queries. In addition, the reference lists of key articles and relevant reviews were manually screened to identify further eligible publications.

### 2.4. Inclusion and Exclusion Criteria

Studies involving adult patients (aged ≥ 18 years) undergoing cardiac surgery were included. Eligible studies reported on one or more of the following: delirium risk factors or pathophysiological mechanisms, screening and/or diagnosis, and prevention and/or management strategies. Only peer-reviewed articles published in English were considered.

Included study designs comprised narrative reviews of appropriate quality, systematic reviews, meta-analyses, randomized controlled trials, and retrospective or prospective cohort studies. Studies focusing exclusively on pediatric populations, those examining non-surgical delirium in medical or ICU patients unrelated to surgery, and case reports were excluded.

All retrieved records were screened independently based on title and abstract for relevance to the topic of postoperative delirium following cardiac surgery. Full-text articles were subsequently assessed for eligibility according to the predefined inclusion and exclusion criteria. In cases of uncertainty, studies were discussed among the authors to reach consensus. The final selection of studies was guided by their relevance, methodological quality, and contribution to the thematic scope of the review.

### 2.5. Data Extraction and Synthesis

Data relevant to postoperative delirium were extracted from the included studies, including study design, population characteristics, delirium assessment methods, risk factors, outcomes, and key findings. Priority was given to high-quality evidence, including systematic reviews, meta-analyses, and randomized controlled trials, while observational studies were included to provide additional context where appropriate.

A narrative thematic synthesis was performed in light of the heterogeneity of the available evidence, with findings organized according to the main themes of the review: epidemiology, psychiatric and biological risk factors, pathophysiological mechanisms, perioperative modifiers, prevention strategies, long-term outcomes, and clinical implications. No formal risk-of-bias assessment tool was applied, as the aim of the review was narrative synthesis; however, study quality was considered during interpretation of the evidence. Where applicable, we distinguish between findings derived specifically from cardiac surgical populations and those extrapolated from broader perioperative or critical care settings.

**Figure 1:**
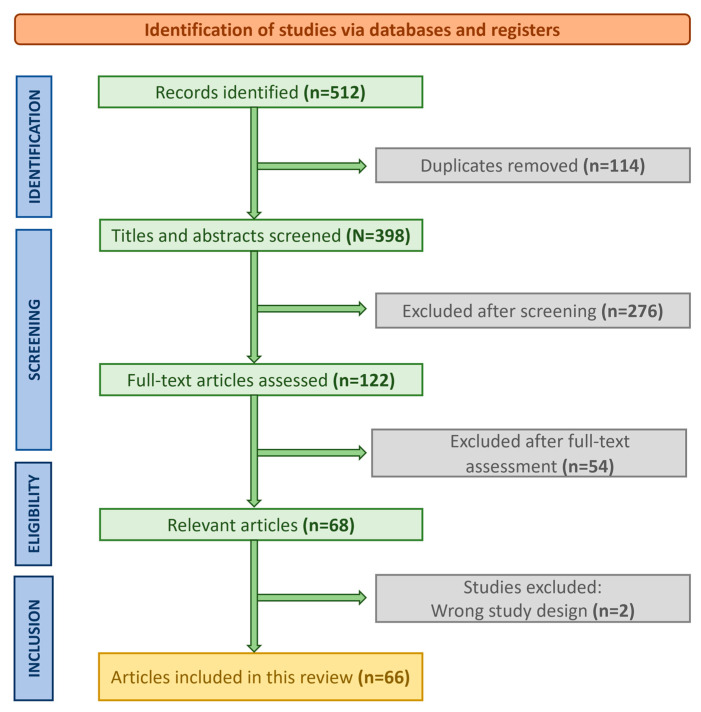
PRISMA flow diagram of study selection. Flow diagram illustrating the process of literature identification, screening, eligibility assessment, and inclusion of studies in this narrative review. A total of 512 records were identified through database searching, of which 398 remained after removal of duplicates. Following title and abstract screening, 122 full-text articles were assessed for eligibility, and 66 studies were ultimately included in the narrative synthesis.

Although this study is a narrative review, a PRISMA-style flow diagram has been included to enhance transparency of the literature selection process (Figure 1).

## 3. Epidemiology and Clinical Presentation of Delirium After Cardiac Surgery

### 3.1. Incidence and Timing

The reported incidence of delirium following cardiac surgery varies considerably depending on the assessment method and frequency of screening. Earlier studies, which relied on non-standardized clinical detection, reported incidence rates as low as 3%. In contrast, more recent studies using validated tools such as the Confusion Assessment Method for the ICU (CAM-ICU), applied multiple times per day, have reported incidence rates ranging from 20% to 50% [2,3].

A 2024 systematic review and meta-analysis including 106 studies reported a weighted pooled incidence of 23% among studies utilizing validated screening tools, although with substantial heterogeneity (I^2^ = 99%) [3]. Delirium most commonly develops within the first 48 to 72 h following cardiac surgery and typically manifests during emergence from sedation and mechanical ventilation in the ICU [19].

In addition, late-onset delirium may occur after transfer from the ICU to a general ward. Such cases are often underdiagnosed due to less frequent monitoring and reduced use of structured assessment tools outside the ICU setting [2].

### 3.2. Clinical Subtypes

Delirium is classified into three clinical subtypes based on psychomotor activity. The hyperactive subtype is characterized by agitation, restlessness, emotional lability, and occasionally aggressive behavior. Although it is the most readily recognized form, it accounts for only approximately 20% of cases following cardiac surgery [20].

The hypoactive subtype, which is the most common form in the cardiac surgical ICU, is frequently underdiagnosed, as patients may appear merely sedated or fatigued. It is characterized by lethargy, reduced psychomotor activity, decreased alertness, and withdrawal [2,20]. A mixed subtype, in which patients fluctuate between hyperactive and hypoactive states, is also commonly observed.

The identification of the hypoactive subtype is particularly important, as it is associated with worse clinical outcomes and increased mortality. Therefore, the use of validated screening tools is important for its systematic detection, rather than relying solely on clinical observation [2].

### 3.3. Diagnostic Challenges

The diagnosis of delirium after cardiac surgery can be challenging due to its multifactorial nature and fluctuating clinical presentation. Patients may appear lucid at the time of assessment yet exhibit delirium at other times, leading to frequent underdiagnosis [2].

In addition, the clinical features of delirium often overlap with those of dementia, depression, and residual sedation, resulting in diagnostic uncertainty, particularly in elderly patients with pre-existing cognitive impairment [21]. For this reason, the use of standardized and validated screening tools is essential.

The 2018 Pain, Agitation, Delirium, Immobility, and Sleep Disruption (PADIS) guidelines recommend routine delirium screening in critically ill patients using validated instruments such as the Confusion Assessment Method for the Intensive Care Unit (CAM-ICU), which demonstrates a sensitivity of approximately 80% and a specificity of around 96% [22,23].

The Intensive Care Delirium Screening Checklist (ICDSC) represents a valid alternative and may offer advantages in detecting subsyndromal delirium [4,24]. Regardless of the instrument used, structured and regular delirium screening—ideally at least once per nursing shift—is essential to ensure accurate detection and timely management.

## 4. Psychiatric and Psychosocial Risk Factors

### 4.1. Pre-Existing Psychiatric Disorders

Pre-existing psychiatric disorders represent an important, yet often underrecognized, risk factor for delirium following cardiac surgery. Among these, depression has been consistently identified as an independent predictor. In a cohort of patients undergoing cardiac surgery, individuals with preoperative depressive symptoms had a 2.19-fold increased risk of developing postoperative delirium compared with those without depression [19,25]. The strength of evidence supporting individual risk factors varies considerably. While some factors, such as pre-existing cognitive impairment and depression, are supported by robust evidence in cardiac surgical populations, others—particularly certain psychosocial variables—are derived from more limited or indirect data and should be interpreted with caution.

This association appears to be particularly pronounced in patients younger than 62 years of age (odds ratio 3.76), suggesting that depression in this group may serve as a strong marker of underlying neuropsychiatric vulnerability [25]. In addition, a systematic review and meta-analysis reported that patients with preoperative depression have nearly twice the risk of developing delirium, with incidence rates of 29% in depressed patients compared to 15% in non-depressed individuals [26,27].

These findings, observed across multiple surgical populations, highlight the importance of routine preoperative screening for depressive symptoms. In contrast, the role of anxiety disorders is less clearly defined. Although patients awaiting cardiac surgery frequently experience elevated levels of preoperative anxiety—reported in up to 31% of patients—this has not been consistently associated with postoperative delirium in multivariate analyses [28,29].

Interestingly, a prospective study identified personality traits related to anxiety, particularly low agreeableness, as independent predictors of delirium. This suggests that psychological factors may indirectly influence delirium risk through mechanisms such as impaired coping capacity and heightened stress reactivity [30].

There is limited evidence regarding the role of bipolar disorder and psychotic disorders in postoperative delirium after cardiac surgery. However, it is plausible that the underlying neurotransmitter dysregulation associated with these conditions may increase susceptibility to delirium in the context of perioperative stress.

### 4.2. Cognitive Vulnerability

Pre-existing cognitive impairment has been consistently identified as a major risk factor for postoperative delirium. Lower preoperative cognitive performance is a stronger predictor of delirium than many other risk factors among older surgical patients, even across the non-demented range of cognitive function [31].

In a cohort of patients aged 70 years and older undergoing major surgery, delirium risk was most strongly associated with preoperative global cognitive function, surpassing variables included in standard delirium prediction models. Lower cognitive reserve was associated with increased vulnerability to perioperative neurological insults [31].

Patients with mild cognitive impairment (MCI) or established dementia represent a particularly high-risk subgroup, as reduced brain resilience limits their ability to compensate for the physiological stressors associated with surgery, anesthesia, and intensive care.

Why some patients develop delirium while others with similar perioperative exposures do not remains an important clinical question. The concept of cognitive reserve provides a useful framework for understanding this variability. Higher levels of education and greater premorbid intellectual engagement are thought to confer a protective effect, whereas lower educational attainment has been identified as an independent risk factor for postoperative delirium in several studies [32].

Assessment of cognitive function in the preoperative setting may therefore play an important role in risk stratification and perioperative care planning.

### 4.3. Substance Use and Withdrawal

Substance use disorders represent a strong and potentially modifiable risk factor for delirium following cardiac surgery. Alcohol use disorder is particularly relevant, and in some cardiac surgical cohorts, recent alcohol use has been identified as the risk factor most strongly associated with postoperative delirium, with one study reporting an odds ratio of 6.11 [33].

Alcohol-related delirium may arise from both the chronic neurotoxic effects of alcohol and the development of withdrawal syndromes during the abstinence associated with hospitalization [34]. Withdrawal-related delirium can occur even at relatively low blood alcohol levels and is thought to result from neuroadaptive changes, including GABAergic tolerance, which lead to central nervous system hyperexcitability upon cessation of alcohol intake. Although this mechanism differs from other forms of postoperative delirium, there is often significant clinical overlap [34].

Through similar GABAergic mechanisms, benzodiazepine dependence is also associated with an increased risk of delirium, and benzodiazepine use is a well-established risk factor in the ICU setting [35]. Nicotine withdrawal, although less well studied in cardiac surgical patients, may also contribute to delirium through disruption of adrenergic and cholinergic pathways.

The identification and appropriate management of substance use disorders in the preoperative period—including the anticipation and prevention of withdrawal syndromes—represent important and potentially modifiable targets for reducing the risk of postoperative delirium.

### 4.4. Psychosocial Factors

Social isolation, low educational level, and preoperative psychological distress have been identified as psychosocial factors associated with an increased risk of delirium. However, the strength of evidence supporting these associations is generally lower than that for psychiatric and cognitive risk factors, and in some cases, extrapolated from non-cardiac surgical populations. As noted previously, low educational attainment has been established as an independent risk factor in several cohorts [32].

Limited social support and social isolation may impair preoperative coping capacity as well as postoperative reorientation and engagement, thereby contributing to the environmental disorientation characteristic of delirium (Table 1). In addition, preoperative stress and fear—particularly related to concerns about mortality or postoperative disability—are common among patients undergoing cardiac surgery. These factors may amplify the neuroendocrine stress response and lower the threshold for delirium [36].

Evidence from clinical trials suggests that family–patient communication may have a protective effect. In one study, patients who received structured family visits in a cardiac surgical ICU had a lower incidence of delirium compared to those without such support [37].

Taken together, these findings suggest that psychiatric and psychosocial vulnerabilities represent an underrecognized component of delirium risk stratification in cardiac surgery. Incorporating structured psychiatric and psychosocial assessment into preoperative evaluation may improve the identification of high-risk patients and facilitate targeted preventive interventions.

## 5. Biological and Pathophysiological Mechanisms

### 5.1. Neuroinflammation and Systemic Inflammatory Response

Neuroinflammation is increasingly recognized as a central mechanism in the development of delirium after cardiac surgery. Cardiopulmonary bypass induces a pronounced systemic inflammatory response through processes such as contact activation, ischemia–reperfusion injury, and endotoxemia, leading to the release of pro-inflammatory cytokines, including TNF-α, IL-6, and IL-8 [38].

In a pivotal study by Rudolph and colleagues (2014), levels of chemokines capable of disrupting the integrity of the blood–brain barrier (BBB) were significantly elevated in cardiac surgery patients who developed delirium compared to matched controls [9]. In particular, chemokines such as C-C motif ligand 2 (CCL2) have been shown to promote leukocyte migration into the central nervous system and contribute to BBB disruption [9].

Disruption of the BBB allows circulating inflammatory mediators to access the brain, resulting in synaptic dysfunction and neuronal injury, ultimately leading to delirium [38,39]. This complex neuroinflammatory cascade provides a mechanistic link between the systemic stress response induced by cardiac surgery and acute brain dysfunction.

### 5.2. Cerebral Hypoperfusion and Microembolization

Reduced cerebral blood flow during and after cardiac surgery represents an important mechanism of brain injury. During cardiopulmonary bypass (CPB), cerebral perfusion depends on pump flow and mean arterial pressure. Inadequate perfusion or impaired cerebrovascular autoregulation may lead to cerebral ischemia [40].

A prospective observational study using near-infrared spectroscopy (NIRS) to assess cerebrovascular autoregulation demonstrated that early postoperative impairment of autoregulation was independently associated with delirium, with an odds ratio of 1.05 for each unit increase in the tissue oximetry index [41]. Furthermore, NIRS-based monitoring of cerebral oxygenation during cardiac surgery with CPB has been associated with a reduced incidence of postoperative delirium, as reported in a systematic review and meta-analysis, suggesting a potential protective effect [42].

Microembolization is another well-established mechanism contributing to brain injury during cardiac surgery. It occurs during aortic manipulation, cannulation, and cross-clamping, particularly in patients with extensive aortic atherosclerosis [10]. These microemboli, which may consist of atheromatous debris, air, or thrombus, can become lodged in the cerebral microvasculature, leading to multifocal ischemia and diffuse cortical dysfunction characteristic of delirium [43].

The duration of CPB has also been consistently identified as a risk factor for delirium, likely reflecting the cumulative embolic burden and prolonged exposure to a non-physiological circulatory state [14].

### 5.3. Neurotransmitter Dysregulation

The neurotransmitter imbalance hypothesis is one of the most widely accepted and extensively studied theories of delirium pathophysiology. A central neurochemical feature of delirium is cholinergic deficiency. Numerous studies have linked reduced acetylcholine synthesis, impaired cholinergic transmission, and exposure to anticholinergic medications with the development of delirium [44].

Acetylcholine plays a critical role in attention, arousal, and cognitive processing, and its deficiency contributes to the inattention and disorganized thinking that are characteristic features of delirium [44,45]. Acute alterations in acetylcholine levels may occur in response to brain insults such as ischemia, inflammation, and systemic stress. Furthermore, overlap between delirium and dementia at genetic, enzymatic, and immunological levels supports the central role of cholinergic dysfunction in both conditions [44].

In addition, dopaminergic excess has been implicated in delirium, with some authors suggesting that the balance between cholinergic and dopaminergic activity may influence clinical presentation, including hypoactive and hyperactive subtypes [46]. Serotonergic dysregulation may also interact with these pathways to precipitate delirium, while gamma-aminobutyric acid (GABA) dysfunction is particularly relevant in the context of sedative use, benzodiazepine exposure, and alcohol withdrawal [46]. An important and often underrecognized contributor to cholinergic dysfunction in delirium is the cumulative anticholinergic burden associated with commonly prescribed medications. A growing body of literature suggests that many drugs not traditionally classified as anticholinergic may still exert clinically relevant anticholinergic effects, particularly when used in combination, leading to an additive or synergistic reduction in central cholinergic activity [47,48]. This cumulative anticholinergic burden has been consistently associated with an increased risk of delirium, cognitive impairment, and adverse outcomes, especially in older and medically complex patients [49]. Notably, medications such as furosemide, although not classically considered anticholinergic agents, have been shown to contribute to overall anticholinergic load through indirect or weak receptor interactions, highlighting the importance of considering total medication burden rather than individual drug classification alone [48,50]. Recognition and minimization of anticholinergic burden in the perioperative period may therefore represent a modifiable target for delirium prevention.

**Figure 2:**
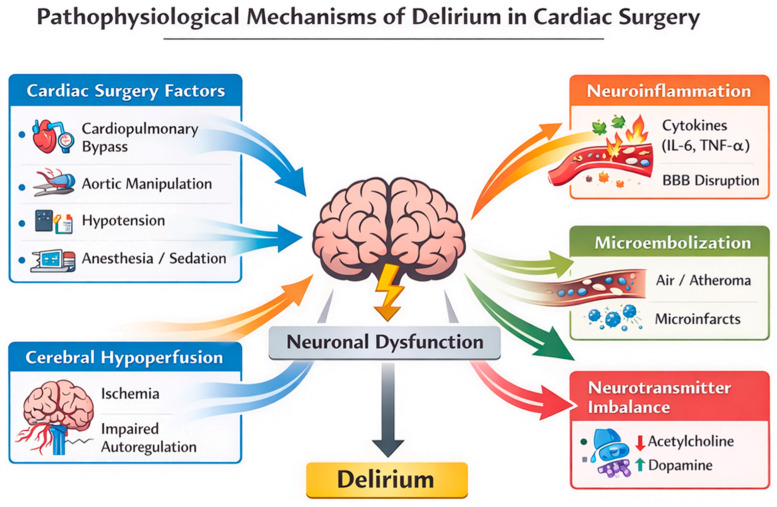
Pathophysiological mechanisms of delirium after cardiac surgery. Cardiac surgery exposes the brain to multiple physiological stressors that may converge to produce acute neuronal dysfunction and delirium. Cardiopulmonary bypass, aortic manipulation, hypotension, and anesthetic exposure can initiate several interconnected mechanisms. These include systemic inflammation with cytokine release and blood–brain barrier disruption (neuroinflammation), cerebral hypoperfusion and impaired autoregulation leading to ischemic injury, microembolization from air or atheromatous debris causing microinfarcts, and alterations in neurotransmitter systems such as cholinergic deficiency and dopaminergic excess. The interaction of these mechanisms results in impaired neuronal signaling and network dysfunction, ultimately manifesting clinically as postoperative delirium.

Ultimately, delirium may be viewed as the clinical manifestation of a final common pathway of neurotransmitter dysregulation that hinders cerebral information processing, regardless of specific upstream insult [46]. The major pathophysiological pathways implicated in postoperative delirium after cardiac surgery are summarized schematically in Figure 2, highlighting the complex interplay between neuroinflammation, cerebral hypoperfusion, microembolization, and neurotransmitter dysregulation, which converge to produce acute neuronal and network dysfunction.

### 5.4. Interaction Between Psychiatric Vulnerability and Biological Stress

The stress–diathesis model provides a useful conceptual framework for understanding why some patients develop delirium while others with similar perioperative exposures do not (Table 2). According to the model proposed by Inouye and Charpentier, delirium arises from the interaction between baseline vulnerability (“diathesis”) and acute precipitating factors (“stress”) [17].

**Figure 3:**
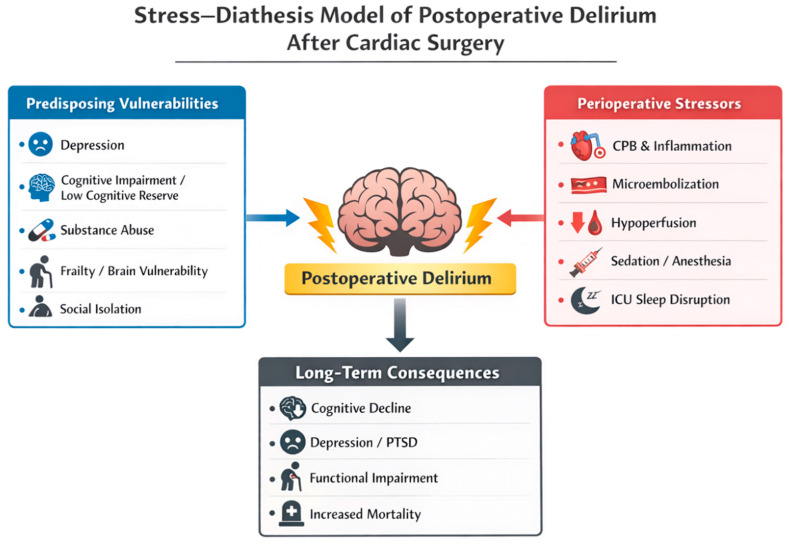
Stress–diathesis model of postoperative delirium after cardiac surgery. Postoperative delirium arises from the interaction between baseline patient vulnerability (“diathesis”) and acute perioperative physiological stressors. Predisposing factors such as depression, cognitive impairment, substance use disorders, frailty, and social isolation reduce neural resilience and increase susceptibility to acute brain dysfunction. During cardiac surgery, stressors including cardiopulmonary bypass-related inflammation, cerebral hypoperfusion, microembolization, sedative exposure, and ICU-related sleep disruption may trigger delirium in vulnerable individuals. The resulting syndrome is associated with important downstream consequences, including cognitive decline, psychiatric morbidity, functional impairment, and increased long-term mortality.

Patients with a high baseline risk—such as those with pre-existing cognitive impairment, psychiatric disorders, or advanced age—may develop delirium in response to relatively minor precipitating insults. In contrast, individuals with greater physiological and neuropsychiatric resilience may tolerate substantial noxious exposures without developing delirium [17,51]. The interaction between predisposing vulnerabilities and perioperative stressors leading to postoperative delirium is illustrated in Figure 3, based on the stress–diathesis model, highlighting how baseline psychiatric and cognitive vulnerability interacts with perioperative physiological insults to trigger delirium and its downstream consequences.

A novel stress–diathesis model of postoperative delirium has been proposed by El-Gabalawy and colleagues, incorporating both baseline vulnerability and intraoperative physiological stressors. In this model, preoperative cerebrovascular reactivity is used as a marker of brain vulnerability, while intraoperative physiological stress represents the precipitating factor. This framework may also have potential applications in individualized risk prediction [52].

The concept of “brain vulnerability” further expands this perspective, reflecting the cumulative burden of neurological deficits arising from psychiatric illness, cognitive decline, and neurodegeneration. This reduced neural resilience limits the brain’s ability to cope with acute physiological insults in a manner analogous to how physical frailty increases vulnerability to adverse health outcomes. Indeed, pre-frail and frail states, as defined by frailty indices, have been independently associated with an increased risk of incident delirium [53].

Recent research has also focused on the role of biological biomarkers in predicting postoperative delirium. Elevated levels of inflammatory markers, including interleukin-6, C-reactive protein, and chemokines such as CCL2, have been associated with an increased risk of delirium following cardiac surgery. In addition, neurodegenerative biomarkers, including tau protein and neurofilament light chain, are increasingly being investigated as indicators of neuronal injury and underlying brain vulnerability. Although these biomarkers are not yet part of routine clinical practice, they may contribute to the development of future risk stratification models.

## 6. Perioperative and Intraoperative Risk Modifiers

### 6.1. Surgical Factors

The risk of postoperative delirium is influenced by the type and complexity of cardiac surgery. Combined procedures, such as coronary artery bypass grafting (CABG) performed alongside concomitant valve surgery, are associated with a higher risk compared to isolated CABG. This is largely due to prolonged cardiopulmonary bypass (CPB) and aortic cross-clamp times [15]. Multiple studies have consistently demonstrated that longer CPB duration is associated with an increased risk of delirium [14,54].

The potential neuroprotective benefit of off-pump CABG compared to on-pump CABG remains a subject of debate. Some studies suggest that off-pump CABG is associated with a lower incidence of postoperative delirium and adverse neurological events [55]. However, large randomized trials such as OCTOPUS and ROOBY, which investigated the long-term neurocognitive effects of avoiding CPB, have not demonstrated consistent benefits [56].

Importantly, off-pump techniques do not fully eliminate the risk of cerebral injury, as factors such as aortic manipulation and hemodynamic instability may still result in cerebral hypoperfusion and neurological insult.

### 6.2. Anesthetic and Sedation Strategies

The risk of postoperative delirium may be influenced by anesthetic management. Processed electroencephalographic indices, such as the bispectral index (BIS), are used to monitor the depth of anesthesia and have been suggested as modifiable factors, with excessive anesthetic depth potentially increasing the risk of delirium [56].

The choice of sedative agents in the ICU is of particular importance. Benzodiazepines have been consistently associated with a higher incidence of delirium compared to other agents. This may be related to their GABAergic effects, disruption of normal sleep architecture, and prolonged half-life in critically ill patients [35,57]. In contrast, dexmedetomidine, an α2-adrenergic agonist, is increasingly used for sedation in cardiac surgical patients.

In a meta-analysis of 31 randomized controlled trials including 5628 patients, dexmedetomidine was associated with a significant reduction in the incidence of postoperative delirium following cardiac surgery (risk ratio 0.61; 95% CI, 0.50–0.75; *p* < 0.001) [58]. Similarly, a double-blind, placebo-controlled trial demonstrated that nocturnal administration of low-dose dexmedetomidine reduced the incidence of ICU delirium, with 80% of patients remaining delirium-free compared to 54% in the placebo group [59].

In addition, high perioperative opioid exposure and prolonged duration of opioid use have been identified as independent risk factors for delirium in patients undergoing cardiac surgery [60].

### 6.3. ICU-Related Factors

The intensive care unit (ICU) environment itself represents a delirium-promoting setting. Critically ill patients in the ICU often experience significant sleep disruption, characterized by reduced total sleep time, frequent awakenings, and suppression of slow-wave and rapid eye movement (REM) sleep [61]. Sleep plays a crucial role in the pathophysiology of delirium; while sleep disruption may contribute to its development, it may also occur as a consequence of delirium, suggesting a bidirectional relationship.

Disruption of circadian rhythms is also common in the ICU and may result from diminished melatonin secretion, impaired light–dark signaling, and the effects of sedative medications [61]. In addition, sensory deprivation—such as the removal of glasses, hearing aids, and familiar environmental cues—can contribute to disorientation in vulnerable patients. Conversely, sensory overload due to continuous monitoring alarms, artificial lighting, and high levels of clinical activity may further exacerbate agitation and confusion [62].

The use of physical restraints represents another important modifiable risk factor. Although often employed to prevent self-harm or accidental removal of invasive devices, physical restraints have been identified as an independent precipitating factor for delirium, with a reported relative risk of 4.4 [17]. Their use, particularly in elderly hospitalized patients, is also strongly associated with the presence of delirium.

## 7. Prevention and Risk Reduction Strategies

### 7.1. Preoperative Interventions

The preoperative phase should include systematic screening and optimization in order to improve the prevention of postoperative delirium. There is evidence to suggest that preoperative psychiatric screening—particularly for depression, anxiety, and substance use disorders—in patients undergoing cardiac surgery is beneficial. Identification of modifiable psychiatric risk factors allows for targeted preoperative optimization [25,36].

Psychological interventions across the perioperative period, including psychoeducation, comprehensive history-taking, cognitive behavioral therapy, anxiety-reduction strategies, and appropriate pharmacological treatment, are recommended as part of a comprehensive care approach in cardiac surgery. However, high-quality randomized evidence demonstrating their direct impact on delirium prevention remains limited [36].

Preoperative cognitive assessment using validated tools such as the Mini-Mental State Examination (MMSE) or the Montreal Cognitive Assessment (MoCA) is important for identifying pre-existing cognitive impairment. This information is valuable for risk stratification and perioperative care planning. In addition, preoperative cognitive “prehabilitation” has shown promise in reducing delirium in older adults undergoing major non-cardiac surgery, although further studies are needed to evaluate its effectiveness in the cardiac surgical population [63].

### 7.2. Intraoperative Strategies

Intraoperative strategies for delirium prevention focus on minimizing cerebral insults associated with cardiac surgery. Cerebral oxygenation can be monitored using near-infrared spectroscopy (NIRS)-based cerebral oximetry, which provides real-time assessment of regional cerebral oxygen saturation and allows for targeted interventions to maintain adequate oxygen delivery [42].

Evidence from several studies suggests that the use of NIRS monitoring during cardiopulmonary bypass (CPB) may reduce the incidence of postoperative delirium. In addition, optimization of mean arterial pressure (MAP) during CPB appears to play an important role. Some studies indicate that higher MAP targets (80–90 mmHg compared to 60–70 mmHg) are associated with a lower incidence of postoperative delirium and less postoperative decline in Mini-Mental State Examination (MMSE) scores [56].

Strategies to reduce cerebral microembolization include minimizing embolic load through careful aortic manipulation, the use of epiaortic ultrasonography to guide cannulation strategies, and the implementation of arterial line filters [43]. Additional modifiable factors include anesthetic choice and titration, with particular emphasis on avoiding excessive anesthetic depth and minimizing benzodiazepine exposure [35].

### 7.3. Postoperative and ICU-Based Interventions

In the ICU, prevention of postoperative delirium is supported by multiple non-pharmacological interventions. Early mobilization, particularly within the first 24 h following cardiac surgery, has been shown to reduce both the incidence and duration of delirium and appears to be safe and feasible in this population [64]. Similarly, a systematic review and meta-analysis of early mobilization programs in critically ill patients demonstrated a significant reduction in delirium incidence and duration [65].

Optimization of the ICU environment is also essential. Nursing activities should be clustered to minimize sleep disruption, and nighttime noise and light levels should be reduced to support circadian rhythm regulation [61]. Regular patient reorientation—such as reminders of date, time, and location—along with the use of visual and hearing aids and the presence of familiar objects, can help mitigate environmental disorientation [17].

Family involvement is a key component of the ABCDEF bundle and has been associated with reduced delirium and improved clinical outcomes. In a randomized trial conducted in a cardiac surgical ICU, structured family–patient communication significantly reduced the incidence of delirium [37,66].

**Figure 4:**
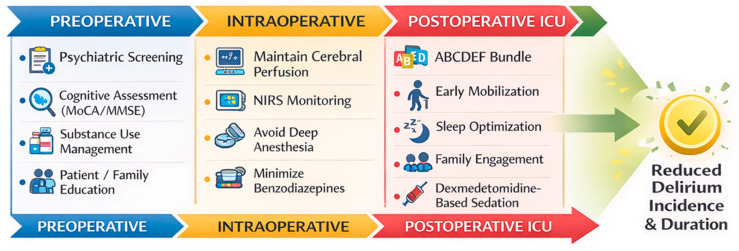
Perioperative prevention strategies for delirium after cardiac surgery. Delirium prevention requires a comprehensive perioperative approach spanning the preoperative, intraoperative, and postoperative phases of care. Preoperative interventions focus on identifying and optimizing patient vulnerability through psychiatric screening, cognitive assessment, substance use management, and patient–family education. Intraoperative strategies aim to minimize cerebral insults by maintaining adequate cerebral perfusion, monitoring cerebral oxygenation using near-infrared spectroscopy (NIRS), avoiding excessive depth of anesthesia, and limiting benzodiazepine exposure. Postoperative prevention in the intensive care unit emphasizes multicomponent interventions such as the ABCDEF bundle, early mobilization, sleep and circadian rhythm optimization, family engagement, and dexmedetomidine-based sedation. Together, these measures contribute to reducing the incidence and duration of postoperative delirium.

The ABCDEF bundle encompasses Assessment, prevention, and management of pain (A); Both spontaneous awakening and spontaneous breathing trials (B); Choice of sedation and analgesia (C); Delirium assessment, prevention, and management (D); Early exercise and mobility (E); and Family engagement and empowerment (F). Multiple large studies have demonstrated a dose–response relationship between bundle implementation and improved clinical outcomes. An umbrella meta-analysis of six meta-analyses including 62,949 patients reported a 50% reduction in ICU delirium (OR = 0.50; 95% CI: 0.39–0.65; *p* < 0.001), as well as significant reductions in delirium duration and in-hospital mortality [66,67]. A summary of perioperative strategies aimed at reducing the incidence and duration of postoperative delirium in cardiac surgery is presented in Figure 4, illustrating a multicomponent and multidisciplinary approach across the preoperative, intraoperative, and postoperative phases of care.

### 7.4. Pharmacological Prevention

Pharmacological approaches to the prevention of postoperative delirium in cardiac surgery remain an area of active investigation. Haloperidol, a first-generation antipsychotic, is one of the most commonly used agents for delirium management and has been evaluated for prophylactic use in several trials. However, an umbrella review of systematic reviews found that haloperidol did not demonstrate a significant benefit in preventing delirium, although it appeared to be safe in the studied populations [68]. Similarly, a meta-analysis comparing haloperidol with placebo failed to show a significant reduction in delirium incidence [69].

In contrast, dexmedetomidine has shown consistent evidence suggesting a potential role in pharmacological delirium prevention in cardiac surgery (Table 3). As noted previously, a comprehensive meta-analysis of 31 randomized controlled trials demonstrated a significant reduction in delirium incidence with dexmedetomidine use (risk ratio 0.61), with findings further supported by trial sequential analysis [58]. However, dexmedetomidine is associated with an increased risk of bradycardia (risk ratio 1.53), highlighting the importance of careful patient selection [58].

Current guideline recommendations, including those from the PADIS guidelines, favor dexmedetomidine over benzodiazepines for sedation in critically ill patients. Nevertheless, multicomponent non-pharmacological strategies, such as those recommended by NICE, remain the cornerstone of delirium prevention [23].

## 8. Long-Term Outcomes and Prognostic Implications

### 8.1. Cognitive Decline and Dementia Risk

Postoperative delirium following cardiac surgery has raised significant concern in both research and clinical settings due to its association with long-term cognitive impairment. A substantial proportion of patients develop postoperative cognitive dysfunction (POCD). Reported rates of POCD range from approximately 11% at 3 months to 41% at hospital discharge [20].

The duration of delirium has been identified as an important predictor of long-term cognitive outcomes, with longer episodes associated with worse global cognitive function at 3 and 12 months. The large prospective BRAIN-ICU study demonstrated that approximately one-third of patients exhibited cognitive performance at 12 months comparable to that of individuals with moderate traumatic brain injury, while one-quarter had scores similar to those seen in mild Alzheimer’s disease [70].

These findings suggest that delirium is not merely a transient state of confusion, but may contribute to or accelerate neurodegenerative processes, potentially leading to persistent neurocognitive disorders, including dementia [6,71].

### 8.2. Psychiatric Sequelae

In addition to cognitive impairment, short-term psychiatric sequelae such as depression, anxiety, and social withdrawal are common following cardiac surgery and critical illness. In one study, 37% of patients reported at least mild depressive symptoms at 3 months, and 33% at 12 months [70]. Depression and anxiety are particularly prevalent among patients who experience ICU admissions complicated by delirium.

Post-traumatic stress disorder (PTSD)-like symptoms are also increasingly recognized in ICU survivors, with approximately 7% of patients exhibiting PTSD symptoms at both 3 and 12 months following ICU admission [70]. A cohort study demonstrated that delirium was independently associated with higher scores on the Impact of Event Scale–Revised, a validated measure of PTSD symptoms, suggesting that the experience of delirium itself may contribute to psychological trauma [72].

**Figure 5:**
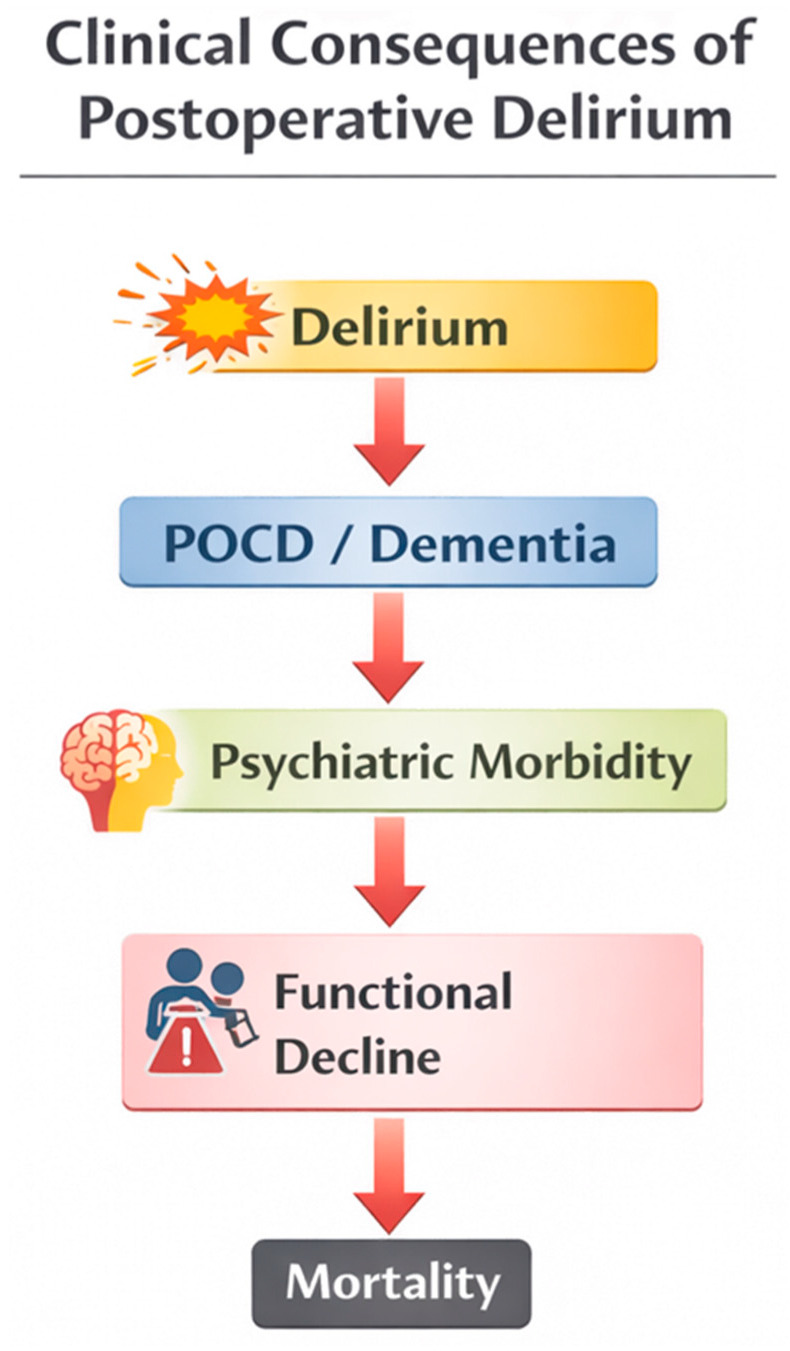
Clinical consequences of postoperative delirium after cardiac surgery. Postoperative delirium is not only an acute neuropsychiatric complication but also a marker of long-term vulnerability to adverse outcomes. Patients who develop delirium after cardiac surgery have an increased risk of persistent cognitive decline and postoperative cognitive dysfunction (POCD), which may progress to dementia in some cases. Delirium is also associated with psychiatric sequelae, including depression, anxiety, and post-traumatic stress disorder-like symptoms. These neuropsychiatric consequences frequently contribute to functional decline, reduced independence, and impaired quality of life. Ultimately, postoperative delirium has been consistently associated with increased short- and long-term mortality.

The constellation of new or worsening cognitive, psychiatric, and physical impairments following critical illness is collectively referred to as post-intensive care syndrome (PICS), which represents a significant public health and socioeconomic burden [21]. The cascade of cognitive, psychiatric, and functional consequences associated with postoperative delirium is illustrated in Figure 5, highlighting its progression from an acute neuropsychiatric syndrome to persistent cognitive impairment, psychiatric morbidity, functional decline, and increased long-term mortality.

### 8.3. Impact on Quality of Life and Survival

Postoperative delirium has a significant impact on both quality of life and long-term survival (Table 4). In a study of 300 patients undergoing cardiac surgery, delirium was independently associated with lower scores across seven of the eight domains of the Short Form-36 (SF-36), a validated measure of health-related quality of life [2].

Functional decline, as assessed by activities of daily living, is also more pronounced in patients who develop delirium. This effect is evident as early as one month following cardiac surgery and may persist up to one year [2]. In addition, postoperative delirium has been consistently associated with increased long-term mortality, as demonstrated in multiple studies within cardiac surgical populations [4,73].

A meta-analysis further demonstrated that postoperative delirium is associated with prolonged mechanical ventilation, extended ICU and hospital length of stay, and increased mortality, highlighting its substantial prognostic impact [73].

## 9. Clinical Implications and Multidisciplinary Management of Delirium Risk

### 9.1. Importance of Psychiatric–Surgical Collaboration

Given the complexity of postoperative delirium, a multidisciplinary approach to perioperative care may be beneficial. Within this framework, liaison psychiatry can contribute to the identification and management of pre-existing psychiatric disorders, assessment of cognitive function, and differentiation of delirium from other neuropsychiatric conditions [36,74]. It is important to note that the level of evidence varies across the factors discussed, and some associations—particularly psychosocial variables—require further validation in cardiac surgical populations. It should also be noted that some of the evidence discussed is derived from broader perioperative and critical care populations and may not be exclusively specific to cardiac surgery, highlighting the need for further dedicated studies in this field. A structured summary of the key risk factors, pathophysiological mechanisms, and preventive strategies discussed in this review is provided in Table 5 to facilitate clinical interpretation.

Psychological interventions delivered throughout the perioperative period—including preoperative psychoeducation, structured psychological assessment, cognitive behavioral therapy, and family-centered interventions—may be implemented within a multidisciplinary framework comprising psychiatrists, psychologists, cardiac surgeons, anesthesiologists, intensivists, nurses, and physiotherapists [36].

To improve patient outcomes, structured delirium care pathways may be beneficial and warrant consideration across the entire perioperative continuum. These pathways should begin in the preoperative outpatient setting and extend through the ICU stay and into post-discharge follow-up.

While the findings summarized in this review provide important insights, they should be interpreted in the context of a narrative synthesis, and conclusions are therefore inherently limited by the heterogeneity and variable quality of the available evidence.

### 9.2. Delirium as a Marker of Brain Vulnerability

Patients undergoing cardiac surgery who develop acute confusion should be systematically evaluated for delirium. Rather than being regarded as a transient and incidental complication, delirium should be reconceptualized as a clinical indicator of underlying brain vulnerability—that is, a manifestation of reduced neural reserve and resilience to surgical stress [52,53].

This conceptual reframing may have important clinical implications. Patients who develop delirium, even if they recover from the acute episode, may remain at increased risk for long-term cognitive decline, dementia, and psychiatric morbidity. Accordingly, these individuals should be identified for structured long-term follow-up and ongoing cognitive monitoring [70].

The occurrence of delirium in younger or otherwise low-risk patients should be considered particularly concerning, as it may signal unrecognized neurological vulnerability or a greater degree of perioperative cerebral injury than anticipated [2].

### 9.3. Implications for Informed Consent and Risk Communication

Delirium is a common complication following cardiac surgery and is associated with significant short- and long-term morbidity, as well as considerable distress for both patients and their families. These factors have important implications for the informed consent process. Inadequate communication regarding the risk of delirium may lead to insufficient patient and family preparedness and potentially adverse outcomes [75,76].

Patients identified as being at high risk based on preoperative assessment may benefit from a detailed and structured discussion of delirium risk. Involving family members in the consent process is particularly important, as their awareness and support may contribute to improved perioperative management and early recognition of delirium. In addition, routine delirium risk screening—potentially incorporating brief screening tools—has been proposed as part of preoperative evaluation [75,77].

A more comprehensive informed consent process that explicitly addresses the neuropsychiatric risks of cardiac surgery, alongside traditional surgical outcomes, may improve patient understanding, support shared decision-making, and enhance overall quality of care.

## 10. Future Directions

Future research on delirium following cardiac surgery should increasingly focus on the development of integrated risk prediction models that combine biological, psychiatric, cognitive, and perioperative variables. Current prediction tools often rely primarily on demographic and surgical parameters, while psychiatric vulnerability and cognitive reserve remain underrepresented despite their well-established association with postoperative delirium risk [17,31,54]. Recent studies have highlighted the potential of multimodal prediction models incorporating neurocognitive assessment, inflammatory biomarkers, and perioperative physiological data to improve individualized risk stratification [78,79]. In parallel, large prospective cohort studies and multicenter registries may further refine predictive algorithms and facilitate the identification of modifiable risk factors across diverse cardiac surgical populations [39,75].

Another important research direction involves the identification of reliable biomarkers and neuroimaging correlates capable of detecting early brain vulnerability or ongoing neuronal injury. Increasing evidence suggests that systemic inflammatory markers, including interleukin-6, C-reactive protein, and chemokines, are associated with delirium development after cardiac surgery [9,38]. In addition, emerging biomarkers of neuronal injury—such as neurofilament light chain (NfL), tau protein, and glial fibrillary acidic protein—have shown promise as indicators of perioperative brain injury and postoperative delirium risk [80,81]. Advances in neuroimaging techniques, including diffusion tensor imaging and functional MRI, may further enhance understanding of structural and functional network disruptions associated with delirium and postoperative cognitive dysfunction [56,82].

Future interventional studies should also explore targeted strategies addressing baseline neuropsychiatric vulnerability. While multicomponent ICU bundles and optimized sedation strategies have demonstrated effectiveness in reducing delirium incidence [23,51,58], relatively few randomized trials have evaluated interventions specifically targeting psychiatric and cognitive risk factors prior to cardiac surgery. Interventions such as preoperative cognitive prehabilitation, structured psychological support, and management of depression or substance use disorders may represent promising approaches for reducing delirium risk in vulnerable populations [19,36,63]. Furthermore, personalized perioperative care pathways integrating psychiatry, anesthesia, and critical care teams may enhance early detection and management of delirium and its associated complications [39,74].

An additional challenge in delirium research is the substantial heterogeneity in outcome definitions, measurement tools, and reporting standards across studies. This variability limits comparability between studies and poses significant barriers to high-quality meta-analyses and evidence synthesis. Increasingly, there are calls for the development of standardized outcome measures or core outcome sets in perioperative and critical care research. While standardization must be balanced against the need for innovation and flexibility in study design, greater consistency in outcome reporting would substantially enhance the ability to compare findings across studies and advance the field. In this context, the role of guideline and consensus groups will be crucial in establishing harmonized frameworks for future research.

Finally, greater attention should be directed toward long-term outcomes and post-ICU recovery in patients who experience delirium following cardiac surgery. Increasing evidence suggests that delirium may represent an early marker of persistent neurocognitive impairment and post-intensive care syndrome [6,69,70]. Longitudinal cohort studies with extended follow-up are needed to clarify the relationship between postoperative delirium, progressive cognitive decline, and the risk of dementia in cardiac surgical populations [83,84]. Such studies may also inform the development of structured post-discharge follow-up programs aimed at cognitive monitoring, rehabilitation, and psychological support for patients recovering from cardiac surgery.

## 11. Limitations

This narrative review has several limitations that should be considered. Although the narrative design allows for a broad synthesis of a heterogeneous body of evidence, it does not provide the same level of methodological rigor as a systematic review or meta-analysis and may be subject to selection bias in study identification and interpretation.

The lack of consensus regarding the definition and assessment of delirium across studies limits the comparability of reported incidence rates and associated risk factors. This issue has also been highlighted in previously published systematic reviews [3]. Furthermore, psychiatric and psychosocial variables remain underrepresented in the cardiac surgical literature. Many large prospective studies and randomized trials—particularly those conducted in recent years—do not systematically collect data on preoperative depression, anxiety, cognitive function, or substance use, thereby limiting the available evidence base.

In addition, this review was restricted to English-language publications, which may have resulted in the exclusion of relevant studies published in other languages.

Another important limitation is the relatively small number of high-quality trials evaluating psychiatric risk modification strategies for delirium prevention in cardiac surgery, representing a significant gap in current knowledge. Finally, many studies are conducted in single centers or specific patient populations, which may limit the generalizability of their findings.

## 12. Conclusions

Postoperative delirium following cardiac surgery is a multifactorial neuropsychiatric syndrome with significant short- and long-term consequences. This review highlights a substantial prevalence—approximately 23% when assessed using validated screening tools—and underlines the important role of psychiatric and psychosocial risk factors, including depression, cognitive impairment, substance use disorders, and low cognitive reserve. These factors remain underrecognized and are not routinely incorporated into standard perioperative risk assessment. Within the framework of the stress–diathesis model, postoperative delirium arises from the interaction between baseline patient vulnerability and perioperative insults such as neuroinflammation, cerebral hypoperfusion, microembolization, and neurotransmitter dysregulation. Effective prevention therefore requires a comprehensive, integrated approach spanning the entire perioperative trajectory.

This approach may include preoperative psychiatric and cognitive assessment, intraoperative optimization of cerebral perfusion and anesthetic management, and postoperative implementation of multicomponent strategies such as the ABCDEF bundle. Among pharmacological options, dexmedetomidine currently has the most consistent evidence supporting its role in delirium prevention in this population. Importantly, delirium may be regarded not only as a transient complication but also as a potential clinical indicator of underlying brain vulnerability. This perspective suggests that structured long-term follow-up may be warranted, as well as ongoing cognitive monitoring, in affected patients. Early identification of at-risk individuals, combined with multidisciplinary prevention strategies integrating psychiatric and surgical expertise, as well as informed consent processes that address neuropsychiatric risks, may contribute to reducing the burden of delirium in cardiac surgical populations. These conclusions should be interpreted with caution, given the narrative nature of the review and the heterogeneity of the underlying evidence base.

## Figures and Tables

**Table 1:** Psychiatric, cognitive, and psychosocial risk factors for delirium after cardiac surgery.

Risk Factor Category	Specific Factors	Proposed Mechanism	Strength of Evidence
Psychiatric disorders	Depression, anxiety, psychosis	Neurotransmitter imbalance, stress response	Moderate–High
Cognitive impairment	MCI, dementia, low cognitive reserve	Reduced neural resilience	High
Substance use	Alcohol, benzodiazepines, nicotine	Withdrawal, GABA dysregulation	High
Psychosocial factors	Social isolation, low education	Reduced coping capacity	Low–Moderate
Prior delirium	History of delirium	Brain vulnerability	High

**Table 2:** Pathophysiological mechanisms of delirium after cardiac surgery.

Mechanism	Cardiac Surgery-Specific Triggers	Neuropsychiatric Effects	Clinical Correlates
Neuroinflammation	CPB, cytokine release	BBB disruption, synaptic dysfunction	Acute confusion
Hypoperfusion	Hypotension, emboli	Ischemia, neuronal injury	Fluctuating consciousness
Neurotransmitter imbalance	Anesthesia, stress	Cholinergic deficiency	Attention deficits
Stress–diathesis interaction	Psychiatric vulnerability	Exaggerated stress response	Prolonged delirium

**Table 3:** Prevention and risk reduction strategies for delirium in cardiac surgery.

Phase	Intervention	Targeted Risk	Evidence Level
Preoperative	Psychiatric screening	Baseline vulnerability	Moderate
Preoperative	Cognitive assessment	Brain frailty	High
Intraoperative	Cerebral perfusion monitoring (NIRS)	Hypoperfusion	Moderate
Intraoperative	Dexmedetomidine	Sedation-related delirium	High
Postoperative	Early mobilization	ICU delirium	High
Postoperative	Sleep optimization	Circadian disruption	Moderate
Postoperative	ABCDEF bundle	Multicomponent prevention	High

**Table 4:** Short- and long-term consequences of delirium after cardiac surgery.

Outcome Domain	Short-Term Effects	Long-Term Effects
Cognitive	Prolonged ICU stay, acute confusion	Persistent cognitive decline, dementia risk
Psychiatric	Anxiety, agitation	Depression, PTSD
Functional	Delayed rehabilitation	Reduced independence
Mortality	Increased in-hospital mortality	Reduced long-term
Healthcare utilization	Longer hospitalization	Higher readmission rates

**Table 5:** Summary of Key Risk Factors, Mechanisms, and Preventive Strategies for Postoperative Delirium After Cardiac Surgery.

Domain	Key Factors	Strength of Evidence	Clinical Implication
Psychiatric	Depression, anxiety, substance use	Moderate–high	Preoperative screening
Cognitive	MCI, dementia, low reserve	High	Cognitive assessment
Psychosocial	Isolation, low education	Low–moderate	Support interventions
Mechanisms	Inflammation, hypoperfusion, emboli	High	Target intraoperative care
Sedation	Benzodiazepines ↑ risk, dexmedetomidine ↓ risk	Moderate–high	Sedation strategy
ICU factors	Sleep disruption, restraints	Moderate	Environmental optimization
Prevention	ABCDEF bundle, early mobilization	High	Standard care pathway

## Data Availability

No new data were created or analyzed in this study.
